# Properties of poly(lactic acid)/walnut shell/hydroxyapatite composites prepared with fused deposition modeling

**DOI:** 10.1038/s41598-022-15622-8

**Published:** 2022-07-07

**Authors:** Xiaohui Song, Wenfang Guan, Huadong Qin, Xingguo Han, Lingfang Wu, Yishen Ye

**Affiliations:** School of Electromechanical Engineering, Guilin University of Aerospace Technology, Guilin, China

**Keywords:** Biomedical engineering, Biomaterials

## Abstract

In this work, fused deposition modeling (FDM) technology was used to prepare poly(lactic acid)/walnut shell/hydroxyapatite (PLA/WS/HA) composite filaments. HA was treated with silane and characterized by Fourier transform infrared spectroscopy (FTIR). The composites were investigated by using simultaneous thermal analyzer, scanning electron microscopy (SEM) and a universal mechanical testing machine. The results showed that incorporating either HA or WS improved the thermal stability and water absorption of PLA, but lowered the tensile and compression strength. Fillers toughened the PLA matrix, resulting in higher tensile elongation and compressive strain. The tensile and compressive strengths of samples significantly dropped after water-immersion for 6 weeks. Finally, scaffolds were manufactured by using FDM. The compression modulus and structural feature of scaffolds indicated that the PLA/WS/HA composites have the potential to be applied in structural parts, such as bone implants.

## Introduction

Environmental pollution, energy crisis and global warming promoted the utilization of biomaterials. Poly (lactic acid) (PLA) originates from starch-containing corps (such as potato and corn), which is prepared through saccharification, fermentation, purification and chemical synthesis of the extracted starch^1^. It has been widely used due to its biodegradable, renewable, biocompatible and processible features. However, its long-lasting biodegradation and hydrophobicity cause it to react difficultly with other substances, limiting its application in the biomedical field^2^. However, these shortcomings can be overcome by incorporating other materials. In recent years, waste biomass has been used in composites with PLA, due to its lighter weight, low cost and nontoxicity, as well as biodegradability and recyclability^3^. Reviews have summarized biomass applied to composite with PLA, including seeds (cotton and milkweed), nutshells (coconut, walnut and peanut shell), basts (flax, ramie, jute hemp), leaves (sisal, banana and palm), stalks (grass, cane, reed, corn, bamboo) and wood fiber^4,5^.

Walnut shell (WS) is the by-product of walnut foods. China's walnut production exceeds 850,000 tons per year, but 67% of walnuts are walnut shells^6^. Most of WS were composted, burned or discarded as waste. Only a few of WS was applied as adsorbents^7^ or fillers for polymers. Monika et al^8^ prepared polypropylene-based composites with walnut shells (10, 20 and 40 wt%). They found that the addition of walnut shell improved the thermal stability of PP/WS composites. Ahlawat et al.^9^ investigated the mechanical properties of polyster/WS composites. They found that with an increase in WS weitht ratio, the tensile modulus of the composites increased, but the flexural modulus and tensile strength decreased. Gürü et al.^10^ produced urea-formaldehyde/WS composite paricleboard and investigated its nonflammability. Beyond the application for polymer, WS can also be used as biological material. FDA has authorized that food containing walnuts could reduce the risk of heart disease^11^. Li et al.^12^ found that WS pigment scavenged DPPH free radicals in vitro, while other extracts from WS reduced the content of malondialdehyde, which had preventive and protective effects against alcohol-induced liver injury.

Meanwhile, several researchers have conducted valuable work on PLA/WS composites. Liu et al.^13^ investigated the mechanical properties of PLA/WS composites. Zheng et al.^14^ found that the tensile strength, impact strength, and elongation at break of PLA/WS composites were enhanced at approximately 0.5 wt% WS. Orue et al.^15^ improved the tensile strength of PLA/WS composites by 50% through incorporating silane into WS. Our research team investigated the thermal and mechanical properties of PLA/WS by using fused deposition modeling (FDM) technology and found that WS enhanced the degradation of PLA. However, the degradation products of PLA decreased the pH of tissues and induce inflammation and autoimmune responses^16^. This issue might be solved by inputting bioceramics^17^.

One of the most extensively applied bioceramics in tissue engineering (TE) is hydroxypatite (HA) due to its similarity to natural bone apatite^18^. HA has excellent osteoconductivity and can promote the osteogenesis, differentiation and proliferation rate of bone cells^19^. The presence of HA on PLA surface strengthened the formation of actin stress fibers and the expression of vinculin in MC3T3-E1 cells^20^, and reduced the accumulation rate of defects^21^. HA is brittle and low biodegradable/bioresorbable^22^, which can be overcome by compositing with PLA. Therefore, PLA and HA are very complementary^16^. HA (40 wt%) improved the bending strength of PLA by 1.8 times^23^, and 15 wt% HA enhanced the crack resistance of PLA during cyclic loading^21^.

However, in TE, apart from the requirement for biomaterials, an ideal bone should also possess a customized geometry to meet personal defects and interconnected pores for cell ingrowth and immigration^24^. FDM (also called 3D printing) is one of the technologies of additive manufacturing (AM) for fabricating parts with complex geometries. It has been extensively used to manufacture PLA/HA and PLA/biomass scaffolds because of its low cost and easy process. Wu et al.^25^ reviewed the FDM of PLA/HA scaffolds. Yeon et al.^26^ 3D printed a bone clip with PLA/HA/silk composites for internal fixation of bone fractures. Sadudeethanakul et al.^27^ fabricated a femoral canine bone fixation plate with PLA-HA composites by using FDM. Saeid et al.^28^ 3D printed a porous PLA/HA scaffold for femoral fracture treatment. Additionally, the low-cycle fatigue behavior^21^, compressive properties, shape memory effect^21^, long-term creep and impact strength^29^ of PLA/HA were studied. Meanwhile, PLA/wood^30,31^, PLA/kraft pine lignin^32^, PLA/jute and flax fibers^33^, PLA/walnut shell^34^ and PLA/macadamia shell^35^ have been investigated. However, few studies have combined PLA/HA with biomass.

This study attempted to fabricate PLA/HA/WS composite filaments by using FDM. The purposes of this study were to modify HA powder with silane, prepare composite filaments, investigate the thermal properties of composites, characterize the mechanical properties and morphologies of the samples with or without water immersion, and inspect the effect of WS (treated WS) and HA content on the porosity and mechanical properties of PLA/HA/WS scaffolds.

## Experiments and methods

### Materials and methods

Poly(lactic acid) (PLA) 2002D was obtained from Nature Works in powder form. Its characteristics provided by the supplier include an average molecular weight of 100,000 g/mol, a specific density of 1.24 g/cm^3^, a melting point of 165.2 °C and a melt flow index of 10 g/10 min.

Walnut shell (WS) was purchased from a local planter in Guangxi, China. Prior to use, it was cleaned to dust, dried in a lab oven and ground into powder by using a high-speed rotary cutting mill (CS-700Y, COSUAI). The WS powders were then treated with 5 wt% NaOH followed by 6 wt% silane (3-amino propyl triethoxy silane, KH550). The details of the treatment of WS powders are described in the literature^34^.

**Figure 1:**
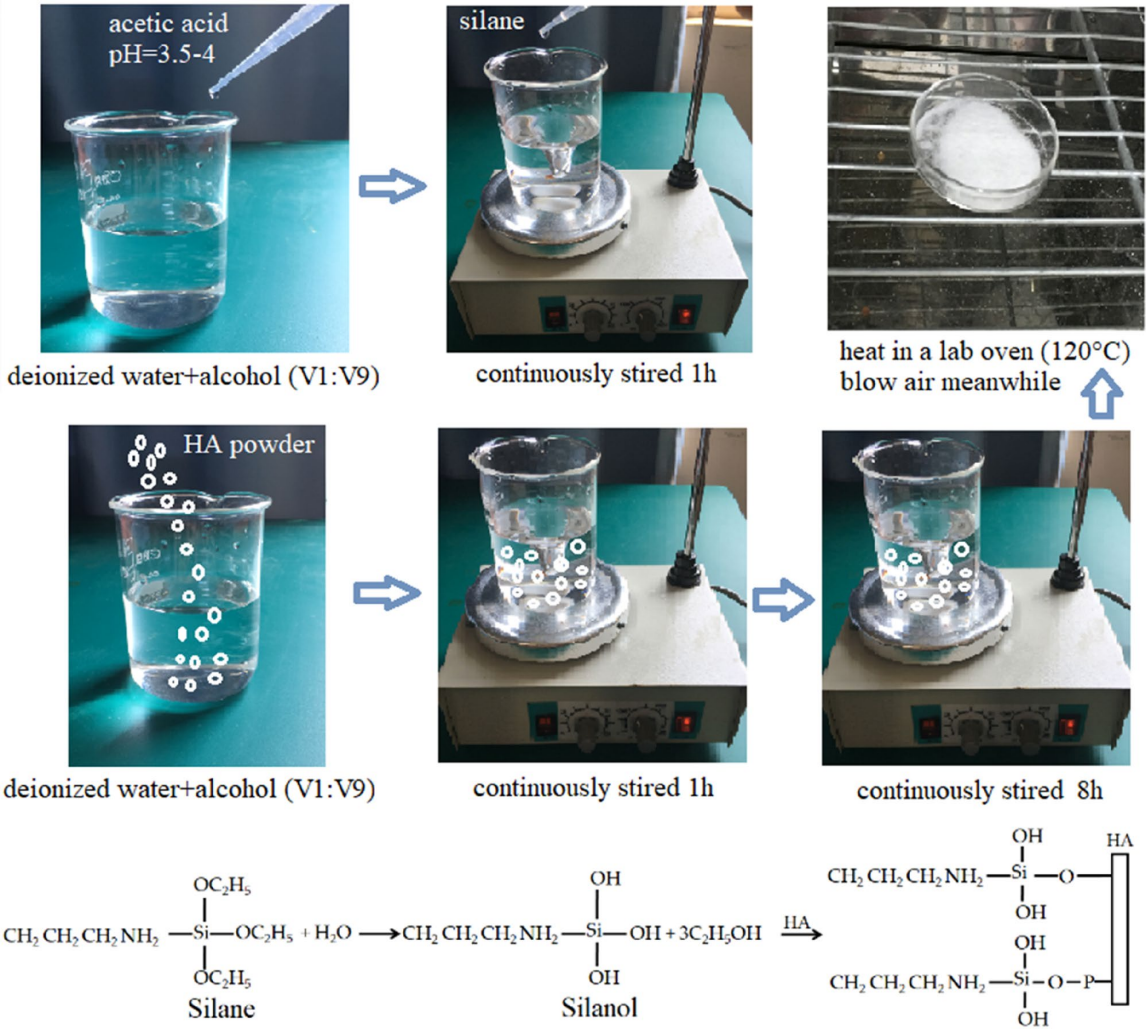
Surface modification of hydroxyapatite.

Hydroxypatite (HA) powder was obtained from Zhejiang Emperor Nanomaterial Co., Ltd. It was plantation-grade and had a density of 3.16 g/cm^3^. The surface treatment process was carried out to enhance the interfacial compatibility with PLA, as shown in Fig. 1. Firstly, a 200 mL solution with 10 vol% deionized water and 90 vol% alcohol were prepared with a pH of 3.5–4, and then silane (KH550) was added at concentrations of 1, 2, 3 and 4 wt%. To hydrolyze the silane completely, the solution with silane was stirred for 1 h by using a magnet stirring apparatus, and then the silanol was produced. On the other hand, the HA powder was put into a 90 vol% ethanol-10 vol% deionized water solution and stirred for 1 h to produce sizing agent. HA sizing agent possessed large specific surface area and had many dangling bonds, which can react with silanol. Secondly, the silane solution and the HA sizing agent were combined and stirred for 8 h continuously. In this step, silanol reacted with –OH, ${\text{PO}}_{4}^{3 - }$ and ${\text{HPO}}_{4}^{2 - }$ groups, and formed strong chemical bond^36^. Afterwards, the residual silane was removed from HA powder by using deionized water. Finally, the treated HA was dried in a lab oven at 120 °C to reduce the moisture content to less than 1 wt%, which was measured with a balance.

### Composite preparation

The treated walnut shell (WS), treated HA and the PLA powders were mixed for 24 h with a planetary ball mill machine (QM–3SP4, Nanjing Yifan Apparatus Co. Ltd., China). Zirconium balls weighing same to the mixture and with diameters of 5 mm and 10 mm were added. After being blended, six kinds of PLA/WS/HA composites were obtained, with the formulations shown in Table 1.

**Table 1:** Formulations of PLA/WS/HA composites.

Samples	PLA (wt%)	WS (wt%)	HA (wt%)
W0H0	100	0	0
W3H3	94	3	3
W3H8	89	3	8
W3H15	82	3	15
W8H3	89	8	3
W15H3	82	15	3

The PLA/WS/HA filaments were prepared on a customized desktop single screw extruder. The parameters of the screw extruder were determined as follows: a screw speed of 20 rpm, a barrel temperature between 165°C and 170 °C, and a die diameter of 1.5 mm. Then, a commercial FDM machine (Allct Yinke, Wuhan, China) was utilized to fabricate these filaments. The processing parameters of the FDM were adopted as follows: a nozzle temperature of 210 °C, a printing speed of 50 mm/s, a layer thickness of 0.06 mm and a nozzle diameter of 0.4 mm. With these parameters, eight dog-bone shaped tensile samples with dimensions of 63.5 mm * 9.53 mm * 3.3 mm were prepared based on the ASTM-638 standard. According to the GBT 1041 standard, eight cylindrical compressive samples (10 mm in diameter and 12 in height) were manufactured. Half of each kind of specimen was used to carry out the tensile and compression tests. Another half of the samples was prepared for the water uptake experiment and combined with the mechanical test. Four cylindrical porous scaffolds with 10 mm in diameter, 12 mm in height and interconnected pores of 1.2 mm in diameter were 3D printed.

### Characterization techniques

A Fourier transform infrared spectrometer (Thermo Nicolet Acatar 360, FTIR) was used to inspect the spectrum data of HA. The KBr disc technique was used for measuring the untreated and treated HA powder. The range of the infrared spectra was chosen between 4000 cm^−1^ and 400 cm^−1^.

The thermal properties of the PLA/WS/HA composite powder were investigated by using a simultaneous thermal analyzer (WCT-121, Beiguang Hongyuan Instrument Co., Ltd, China). The test was carried out under a nitrogen flow rate of 20 mL/min. Approximately 12 mg composite powder per sample was placed in a ceramic crucible. A program for the sample testing was set as follows: heating from room temperature to the final temperature of 500 °C at 10 °C/min heating rate, holding for 5 min, and then cooling to the room temperature. With testing results, the thermogravimetry (TG) data, glass transition temperature ($T_{g}$), melting point ($T_{m}$) and melting enthalpies ($\Delta H_{m}$) were obtained.

To examine the function of water uptake on the mechanical properties and the morphologies of composites, a water-resistance test was carried out. Four of both the cylindrical and dog-bone FDM samples were dried in a lab oven at 50 °C for 24 h. Then, the samples were immediately weighed (W_1_) by a scale and immersed in deionized water for 6 weeks. After each week, the samples were taken out from water, dried surface with tissue, and then measured (W_2_). The moisture content (*Wa*) of the samples was calculated with the following equation:

1 $$Wa = \frac{{W_{2} - W_{1} }}{{W_{1} }} \times 100\%$$

Two types of universal testing machines were adopted. One (Germany Zwick Roell, 2kN) was used to carry out the tensile property at 0.05 mm/s testing speed. Another (Model 8800, Instron Canton, MA, 500 kN) was applied to the compression testing, with a crosshead speed of 1 mm/min. The preload for both testing was 0.1 N. For each, the average value was taken from four measurements.

The porosity of the scaffold was calculated by this equation^37^:

2 $$Porosity = 1 - \frac{{\rho_{0} }}{{\lambda_{PLA} \times \rho_{PLA} + \lambda_{WS} \times \rho_{WS} + \lambda_{HA} \times \rho_{HA} }}$$

where $\uplambda _{{{\text{PLA}}}}$, $\uplambda _{{{\text{WS}}}}$ and $\uplambda _{{{\text{HA}}}}$ represent the weight ratio of PLA, WS and HA in composites; $\uprho _{{{\text{PLA}}}}$, $\uprho _{WS}$ and $\uprho _{{{\text{HA}}}}$ denotes the theoretical density (g/cm^3^), $\rho_{PLA}$ and $\rho_{HA}$ equals to 1.24 g/cm^3^ and 3.16 g/cm^3^, respectively.$\uprho _{{{\text{WS}}}}$ is 1.079 g/cm^3^ measured by pycnometer method; $\rho_{0}$ denotes the apparent density computed with:

3 $$\rho_{0} = m_{0} /V_{0}$$

where $m_{0 }$ is the mass (g) of composite sample obtained with a balance and *V*_*0*_ denotes the volume (mm^3^) of obtained with a vernier caliper.

A scanning electron microscope (SEM, VEGA3, TESCAN) equipped with an energy dispersive spectroscopy (EDS) was applied to characterize the tensile fracture surface of the sample. Prior to characterization, the fracture surface was gold coated with a current of 20 mA for 3 min.

## Results and discussions

### Modification of HA

#### The morphology of untreated and treated HA powder

**Figure 2:**
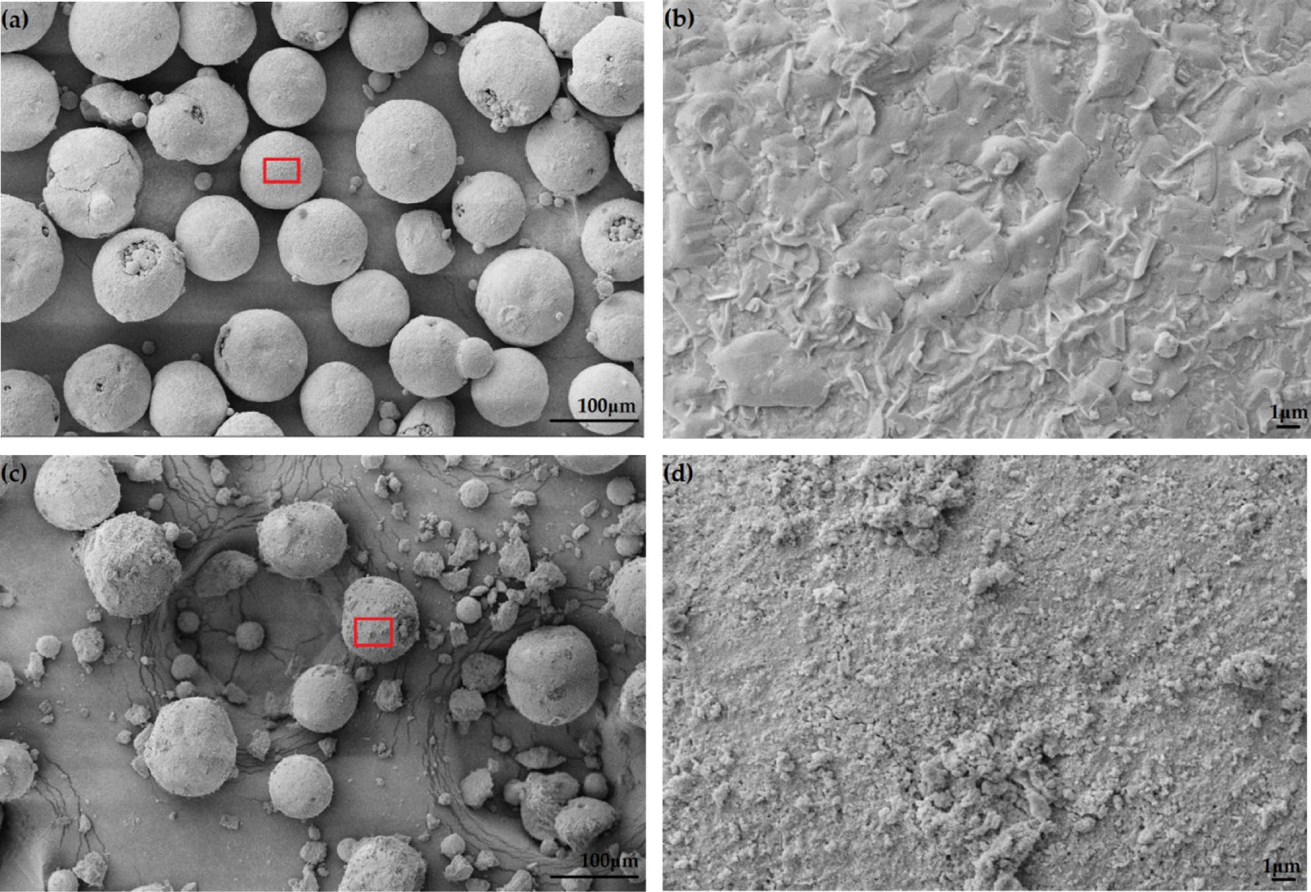
Morphology of HA: (**a**) untreated, (**b**) magnified image of the red rectangle in (**a**, **c**), treated and d, magnified image of the red rectangle in (**c**).

Figure 2 shows the morphology of untreated and treated HA particles, which showed a near-spheroidal shape. The particles consisted of larger powders with diameters of 50–100 μm and smaller powders with diameters of 5–10 μm. After the treatment with silane (3 wt%), the surface of HA was rougher (Fig. 2d) than that of the untreated HA (Fig. 2b). There was a layer of matter on treated HA surface, indicating the chemical reaction or physical tangling of silane with HA (Fig. 2d). Silane molecules evenly distributed on the surface of HA with few aggregations, which might weaken the interfacial compatibility with PLA.

#### FTIR of HA

**Figure 3:**
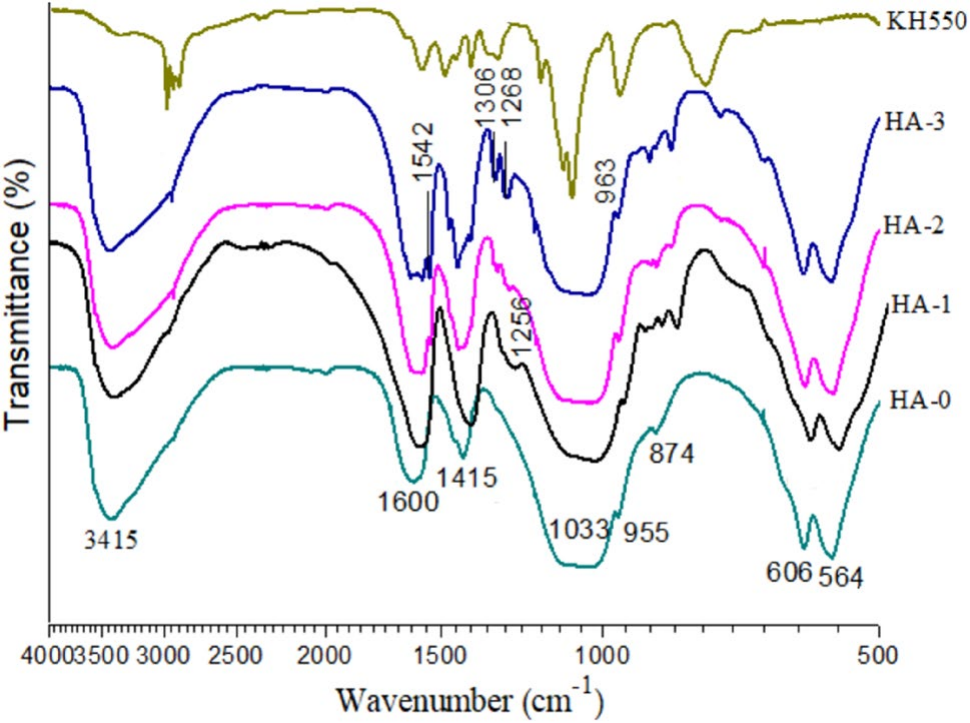
FTIR spectra of HA treated with various concentrations of 0 wt% (HA-0), 1 wt% (HA-1), 2 wt% (HA-2) and 3 wt% (HA-3) of KH550.

Figure 3 shows the FTIR spectra of HA modified with various concentrations of silane. From the spectrum of untreated HA (HA-0), several bands can be seen, including^38^: peaks 606 cm^−1^ and 564 cm^−1^ representing the bending vibration of ${\text{ PO}}_{4}^{3 - }$, bands 955 cm^−1^ and 1033 cm^−1^ contributing to the stretching vibration of ${\text{ PO}}_{4}^{3 - }$, bands 3415 cm^−1^ and 1600 cm^−1^ due to the vibration of absorbed H_2_O, and bands 1415 cm^−1^ relating to the ${\text{CO}}_{3}^{2 - }$ group. After modification, the band at 955 cm^−1^ shifted to its left at 963 cm^−1^, and a new shoulder band appeared at 1256 cm^−1^ (HA-1), representing the group of Si–O–P that reacted between ${\text{PO}}_{4}^{3 - }$ and the group Si–O of silanol^36^. When the concentration of HA was more than 2 wt%, the shoulder bands at 1268 cm^−1^, 1306 cm^−1^, 1390 cm^−1^, 1542 cm^−1^ and 2935 cm^−1^ were all from the KH550 group, contributing to the –OH, Si–O, –NH and –CH_3_ groups, respectively. These bands indicated that silanol deposited on surface of HA (Fig. 2d). Therefore, comprehensively considering the morphology of 3 wt% silane-modified HA, a 2 wt% concentration of silane was sufficient for the treatment of HA and should be determined for further experiments.

### The thermal peoperties of the PLA/WS/HA composites

#### The DSC properties

**Figure 4:**
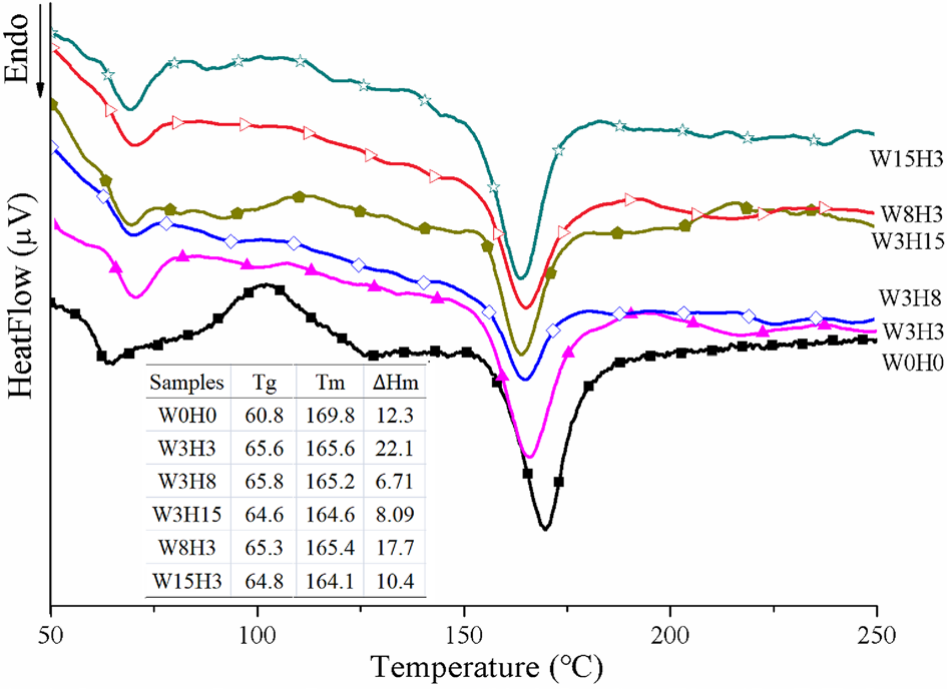
The DSC curves of the composites PLA/WS/HA.

Figure 4 gives the DSC curves of PLA/WS/HA composites. All the samples possess two endothermic phase transformations. One contributed to the glass transition ($T_{g}$, °C), and another referred to the melting procedure ($T_{m}$, °C). However, only the curve of W0H0 (PLA) showed an obvious exothermic phase transformation, which was the cold crystallization of PLA. When compared to that of W0H0 matrix, $T_{g}$ of composites was improved, but Tm decreased. In our previous work, NaOH/silane treated WS (3 wt%) lowered $T_{g}$ and promoted Tm of PLA^34^, indicating that the input of HA took the key effect in this work. HA particle is rigid, resulting in the stiffness increase of PLA molecular chains and a higher $T_{g}$. During ball-grinding, the growing content of particles increased the opportunity of aggregation, leading to a weak compatibility and enhanced mobility of PLA molecules. Meanwhile, according to the infiltration-adsorption theory, the achievement of PLA coating on particles relies on the intermolecular forces, which functions only when the distance between molecules is smaller than 0.5 nm. Therefore, aggregation of particles widened the distance between molecules and loosened molecular chains of PLA, thus lowered the melting temperature of PLA.

The effect of HA and WS on $\Delta H_{m}$ was significant. A small amount of HA and WS improved the $\Delta H_{m}$ of PLA composites from 12.3 J/g (W0H0) to 22.1 J/g (W3H3). However, the further increase of either HA or WS dramatically lowered $\Delta H_{m}$, which was 8.09 J/g and 10.4 J/g at 15 wt% of HA and WS, respectively. This result probably contributed to two reasons: the agglomeration of autopolymerized siloxane on the surface of HA led to poor interfacial compatibility and free molecules; the aggregation of WS and HA particles themselves hampered the integration with PLA matrix. These two reasons caused PLA molecules to melt easily by absorbing less heat.

In other studies, PBLG-g-modified HA increased the Tg but decreased the Tm of PLLA/HA composites. Kothapalli et al.^39^ and Bleach^40^ also obtained an increase in $T_{g}$ by increasing the content of calcium phosphate filler. Wilberforce et al.^41^ observed that nanosized HA was more effective than microsized HA for enhancing $T_{g}$ and cold crystallization. In this study, the size of HA and WS was micro grade, the contact area with PLA was small, and there was no more effective nucleating agent than nanoparticles^41^, so the cold crystallization of PLA/WS/HA composites can be ignored.

#### The thermal stability

**Figure 5:**
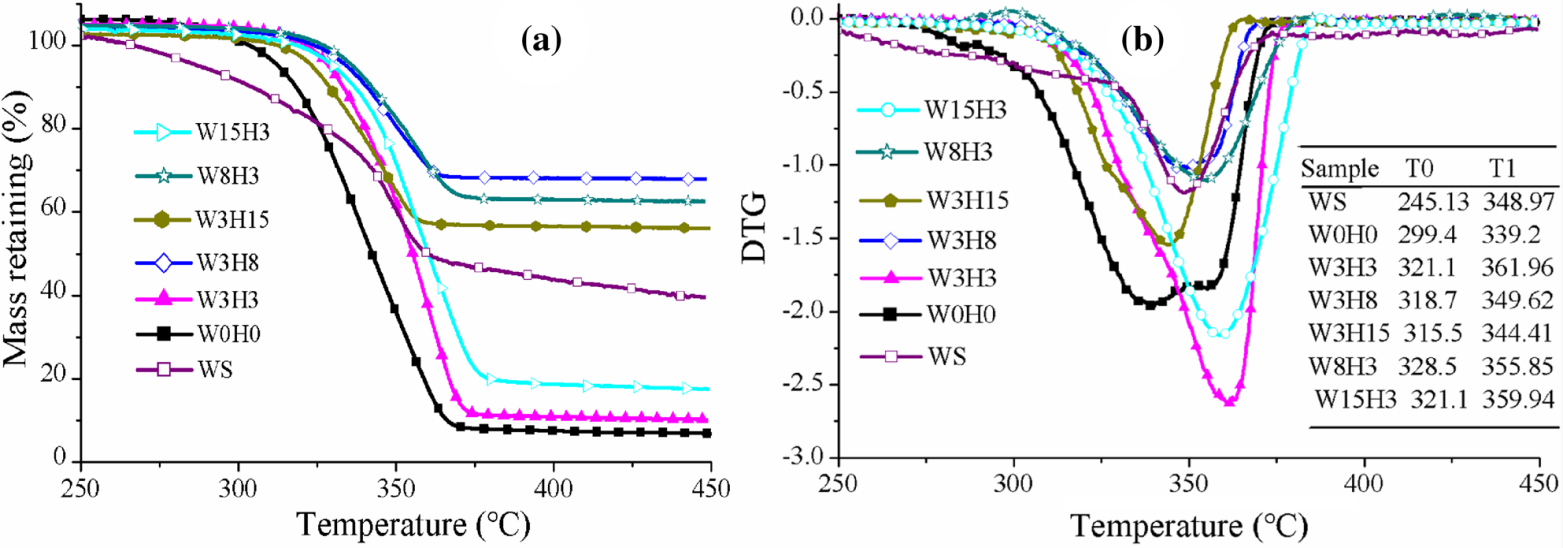
TG curve (**a**) and DTG curve (**b**) of PLA/WS/HA composites: T0, onset temperature of degradation, T1, temperature at maximum degradation rate.

Figure 5 depicts the TG and DTG curves of the WS and PLA/WS/HA composites. All samples showed a single degradation stage during the heating temperature range. The W0H0 sample (neat PLA) possessed the lowest degradation temperature, with a T0 of 299.4 °C and T1 of 339.2 °C, compared to its composites. After incorporating WS and HA, both TG and DTG curves of composites shifted to higher temperature, indicating that PLA/WS/HA composites were more thermally stable than neat PLA.

WS began to degrade at 245.13 °C, which was much lower than that of PLA, indicating that WS reduced the thermal stability of PLA. Our previous work has proved that WS slightly decreased the T0 of PLA from 291.04 to 286 °C^34^. Zhang et al.^42^ found that nano-HA lost only 13% mass at 800 °C, showing a strong thermal stability. Therefore, under the comprehensive effect of HA and WS, the thermal stability of PLA was enhanced.

The varying content of HA and WS influenced the thermal stability of PLA. A small number of particles pushed the T0 and T1 of composites to climax values of 328.5 °C (sample W8H3) and 361.96 °C (sample W3H3). The increase of HA weight ratio decreased T0 and T1 from 321.1 °C and 361.96 °C (sample W3H3) to 315.5 °C and 344.41 °C (sample W3H15). While the rise of WS had less effect on thermal stability than HA did, leading to a moderate drop in T1 from 361.96 °C (sample W3H3) to 355.85 °C (sample W15H3). The results indicated that either the redundant HA or WS particles probably aggregated, weakening the interfacial compatibility between PLA and fillers and loosening the packed PLA molecular chains.

### The mechanical properties of the PLA/WS/HA composites

**Figure 6:**
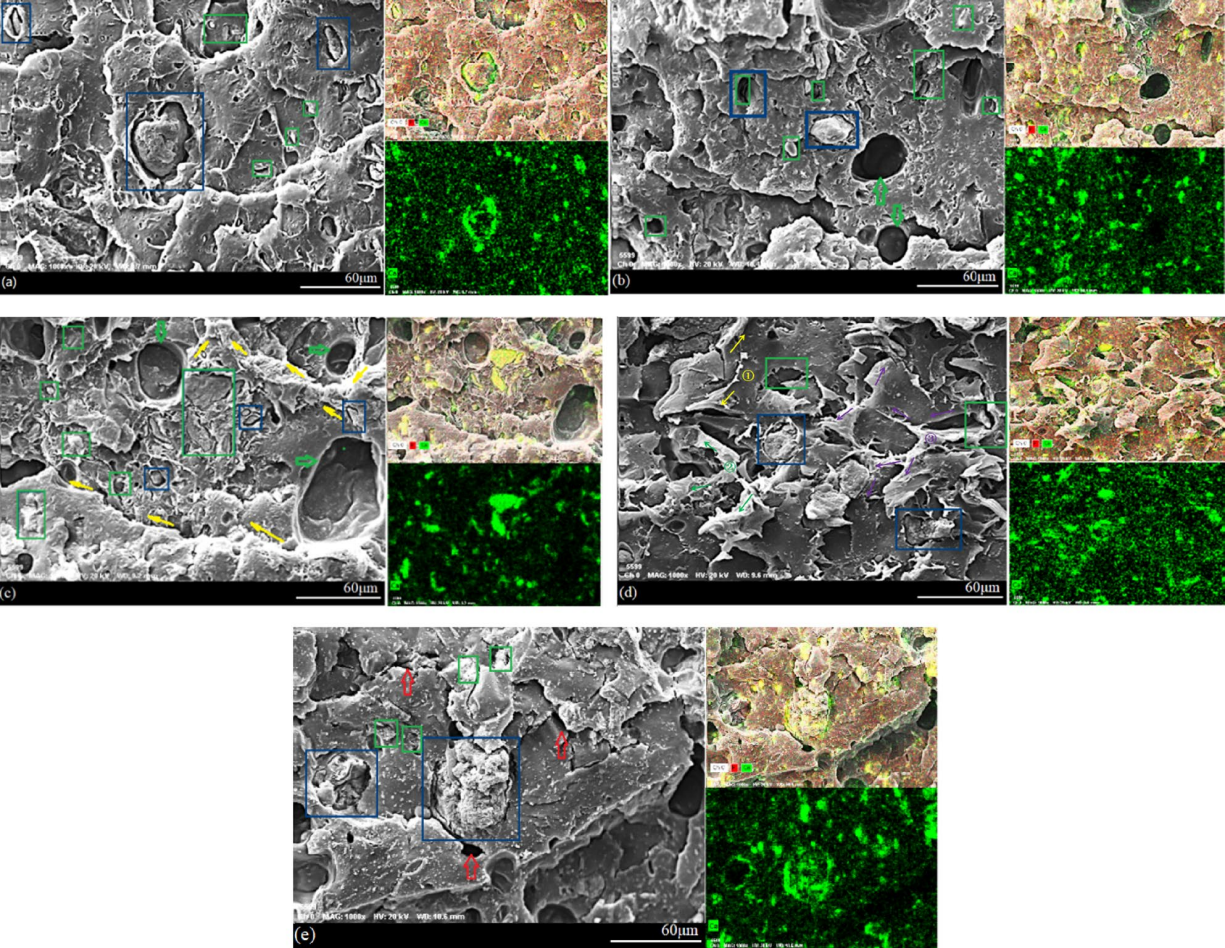
Micrographs of the tensile fractured surface of the PLA/WS/HA composites: (**a**) W3H3, (**b**) W3H8, (**c**) W3H15, (**d**) W8H3 and (**e**) W15H3.

The tensile (ultimate) and compression (yield) properties of PLA/WS/HA composites are given in Table 2. It can be seen that either WS or HA weakened the strength of PLA. The increasing of HA content (0–15 wt%) reduced gradually the tensile strength of PLA from 57.32 MPa (W3H3) to 37.64 MPa (W3H15). This result contributed to the affinity between HA and WS (shown in Fig. 6), which interfered with the interficial compatibility between particles and PLA matrix and hampered the force transfer. The aggregation of HA in sample (shown in Fig. 6c) further lowered the tensile strength.

**Table 2:** Tensile and compression properties of the PLA/WS/HA composites.

Samples	Tensile (ultimate)		Compression (yield)	
	Strength (MPa)	Strain (%)	Strength (MPa)	Strain (%)
W0H0	57.32 ± 0.67^43^	3.00 ± 0.15^43^	92.17 ± 0.89^44^	4.56 ± 0.16^44^
W3H3	45.16 ± 1.05	3.29 ± 0.07	86.05 ± 1.74	5.24 ± 0.10
W3H8	42.76 ± 0.12	3.79 ± 0.01	85.29 ± 0.48	6.42 ± 0.21
W3H15	37.64 ± 0.01	3.95 ± 0.02	75.74 ± 2.44	6.23 ± 0.19
W8H3	51.49 ± 0.61	6.76 ± 0.23	86.19 ± 1.76	4.97 ± 0.16
W15H3	34.24 ± 0.64	2.88 ± 0.06	81.48 ± 1.15	5.00 ± 0.12

Adding HA firstly enhanced the tensile strain slightly from 3% (W3H3) to 3.95% (W3H15). Figure 6a,b showed that HA particles distributed uniformly in the PLA, emboding a good compatibility and enhancing the tensile strain of PLA. This result indicated that the extrusion and 3D printing processes rearraged PLA molecular chains and improved the compatibilty between HA and PLA. However, 15% weight ratio of HA created aggeragation (shown in Fig. 6c) and further reduced tensile strain.

The varing ratio of WS did a major effect on tensile properties, which increased both the tensile strength and strain from 45.16 MPa and 3.29% (W3H3) to 51.49 MPa and 6.76% (W8H3), followed by a drop to 32.24 MPa and 2.88% (W15H3). The results indicated that a proper content of WS was beneficial to the tensile properties of the composites. Too much WS aggregated in the PLA matrix and caused cracks (Fig. 6e), which hampered the force transfer and prevented the unfolding and orientation of the PLA molecule chains. In other literature, the optimum weight ratio of WS was 10%^13,34^, which was near to the result (8%) in this work.

Compared with PLA, composites possessed inferior compression strength and superior strain. Meanwhile, the effect of the variation in WS and HA content was moderate and had the similar tendensy with tensile properties.

### Micrographs and toughing mechanism of the tensile fractured surface of PLA/WS/HA composites

The tensile fractured surface of PLA/WS/HA composites is shown in Fig. 6. EDS images provided the distribution of calcium atom (Ca, shown in green), which distributed uniformly in the PLA matrix and embedded in the matrix without a gap with the matrix (green rectangle), indicating an excellent HA-PLA adhesion. However, the incorporated WS (blue rectangle) had a significant effect on the morphology, showing a strong affinity with the HA particles. Therefore, HA particles aggregated around WS granules (Fig. 6a) and caused gaps between PLA and WS. These gaps hampered the force transfer during the tensile process. As HA content increased, the compatibility between PLA and WS was further aggravated and caused pits on fracture surface (Fig. 6b,c, green hollow arrow). In Fig. 6c, few of HA granules aggregated, which would affect the tensile properties of the composite. While keeping HA content at 3 wt% but increasing WS content to 8 wt%, the morphology of the composite was obviously improved. Although a small gap existed between PLA and WS, both WS and HA were distributed uniformly on the PLA matrix without aggregation (Fig. 6d). However, increasing neither WS content nor HA content improved the quality of fracture surface. The WS granules aggregated and even caused cracks in the PLA matrix (Fig. 6e, red arrow).

The elongations of samples W3H15 and W8H3 were relatively larger than that of the other samples (Table 2), indicating that these samples were toughened. Normally, there are two mechanisms to enhance the fractural toughness of polymer composites by fillers, including debonding or pull-out and crack deflection. Firstly, when the interfacial compatibility between PLA and WS or HA was not strong, WS or HA was debonded or pulled out from the PLA matrix, resulting in gaps (green and blue rectangle) and pits (green hollow arrow). This kind of debonding or pull-out dissipated the tensile energy to toughen the composite. Secondly, crack deflection was another way to toughen the composite. When the crack tips encountered the filler, they then turned to other directions or penetrated the filler. The crack deflection increased the fracture area and further increased the energy consumption. In Fig. 6c,d,e, the crack (yellow, green and purple solid arrows) deflected several times and dispersed on the whole fractural surface and then consumed energy to toughen the matrix PLA. However, the cracks that passed through the WS particles (Fig. 6e, blue rectangle) did not improve the toughness of the PLA matrix, resulting in poor elongation (2.88%).

### Function of water uptake on the properties of PLA/WS/HA composites

#### Relationship between immersion time and moisture content of composites

**Figure 7:**
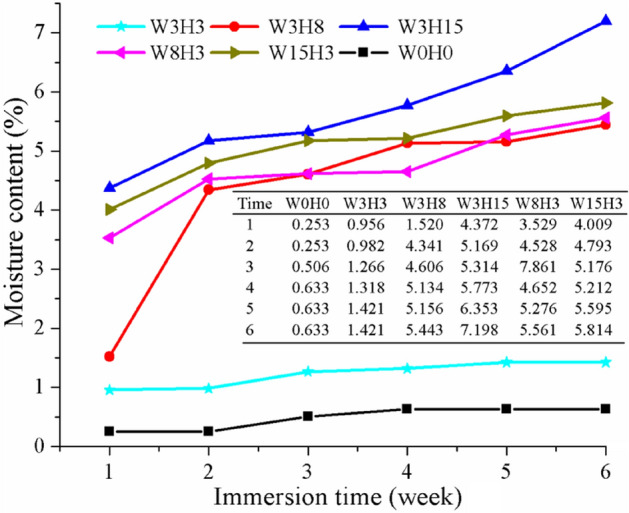
The moisture content of PLA/WS/HA composites as a function of soaking time in water. * Data of W0H0 was refered from our previous work^43^.

To investigate the water uptake of PLA/WS/HA composites, the samples were soaked in deonized water for 6 weeks, with the mositure content recorded and shown in Fig. 7. All the moisture contents grew as the immersion time increased. PLA possessed the lowest moisture content and kept 0.633% after being soaked for 6 weeks, showing a relative hydrophobility. While, a small amount of HA and WS (3 wt%) only increased the final moisture content to 1.421%.

As the increase of HA or WS, the moisture content curves of the composites moved upward. Sample W3H15 had the highest moisture content of 7.198%, followed by sample W15H3 (5.814%). The initial week functioned mostly, resulting in around 3–4% moisture content as for W3H15, W8H3 and W15H3. The results can be attributed to several reasons^45^: (1) both HA and WS possessed OH polar groups that can react with H_2_O and result in the formation of hydrogen bonds; (2) the existence of WS and HA in the PLA matrix rearranged the PLA molecular chains, resulting in a free volume of PLA and more room for water filling; (3) the silanol (Si–O–H) groups coated on the surfaces of HA and WS absorbed water onto its surface; and (4) the aggregation of the redundant WS and HA particles in the PLA matrix loosened the PLA molecular chains and created microvoids in the matrix, resulting in additional water uptake. The water absorbed in the matrix inevitably influenced the mechanical properties of PLA/WS/HA composites, which required further investigation.

#### Tensile and compression properties of the PLA/WS/HA composites after water immersion

The mechanical properties of the water-immersed PLA/WS/HA composites are given in Table 3. The tensile properties were obviously reduced, when compared to those in Table 2. However, the compression strength of the water-immersed samples showed a moderate drop from that of the non immersed ones. The tensile strength of sample W3H15 dropped to less half of its unsoaked ones. However, the reduction in tensile strength of W15H3 was relatively moderate. This result indicated that 15 wt% HA affected the water uptake strongly than 15 wt% WS. As analyzed previously, the samples with a higher HA content possessed a higher moisture content. After being water-soaked for 6 weeks, there were a mass of voids and cracks in the composite, which strongly hampered the force transfer under the tensile force. Noteworthily, Either HA or WS content increased from 8 % to 15 wt%, both the tensile and compression strengths declined slightly. This result was very beneficial to the application of samples in TE, where composites with higher WS and HA content would degrade quicker with a slight decline in their resistance to tension and anti-pressure capability.

**Table 3:** Mechanical properties of the PLA/WS/HA composites after being immersed in deonized water for 6 weeks.

Sample	Tensile (fracture)		Compression (yield)	
	Strain (%)	Strength (MPa)	Strain (%)	Strength (MPa)
W0H0	3.45 ± 0.18	56.12 ± 1.36	5.88 ± 0.29	86.49 ± 0.67
W3H3	3.11 ± 0.27	34.11 ± 0.99	5.87 ± 0.16	87.39 ± 3.05
W3H8	3.36 ± 0.61	19.12 ± 1.04	6.86 ± 0.41	75.81 ± 2.61
W3H15	2.71 ± 0.45	18.48 ± 0.34	6.11 ± 0.08	73.06 ± 2.27
W8H3	3.47 ± 0.03	23.04 ± 0.15	5.12 ± 0.24	68.16 ± 3.04
W15H3	3.62 ± 0.19	22.96 ± 0.13	6.49 ± 0.62	63.28 ± 2.00

#### Microstructures of PLA/WS/HA composites after being water-immersed

**Figure 8:**
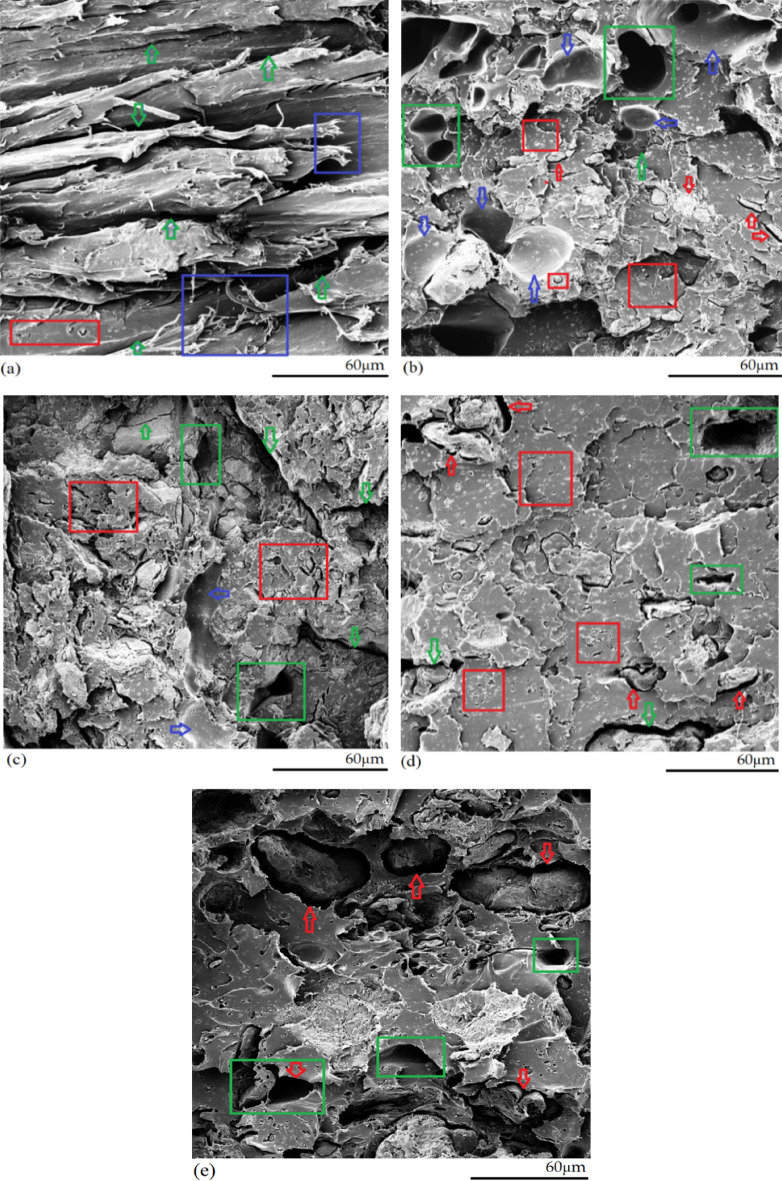
Morphology of the tensile fractured surface of the PLA/WS/HA composites after being immersed in deonized water for 6 weeks: (**a**) W3H3, (**b**) W3H8, (**c**) W3H15, (**d**) W8H3 and (**e**) W15H3.

The tensile fracture surfaces of the water-immersed PLA/WS/HA composites are shown in Fig. 8. After being water-soaked for 6 weeks, the quality of the surface was much worse than that of the unsoaked ones, which showed various defects on the surface. The incorporation of HA or WS had an obvious effect on the quality of morphology. In the case of low HA and WS content (W3H3, Fig. 8a), the morphology quality was fairly good. The extended polymer (blue rectangle) proved a plastic fracture. However, on the surface, there were many cracks (green arrow) due to water uptake, which weakened the sample. As HA content increased, the sample gradually became loose and porous (Fig. 8b,c). The gaps (red arrow) between HA particles and the PLA matrix became larger, which further led to cracks. Some particles were pulled-out and caused voids (green rectangle) and pits (blue arrow). A mass of micropores (red rectangle) were observed, which might contribute to the dissolution of HA particles in water. When WS content in the composite increased, the gaps between WS particles and the PLA matrix became even larger than those with higher HA content. It seemed that the WS particles separated the PLA matrix into pieces, resulting in cracks and voids (Fig. 8e). Those voids, gaps and cracks strongly affected the tensile strength and lowered the ressistance to tension.

### The manufacturing of the PLA/WS/HA composite scaffolds

The scaffolds of the PLA/WS/HA composites were manufactured by using FDM. The compression properties of the scafflods at the yield point are given in Table 4. The scaffolds possessed a cylindrical shape with a diameter of 10 mm and height of 10 mm and combined interconnected pores with a diameter of 1.2 mm. The literature reported that when the pore size was larger than 200 μm, the pore was beneficial to bone ingrowth^46^. The scaffold had a long yield plateau, indicating an excellent anti-forming capacity, as presented in the compression curve of sample W3H15. Table 4 shows that WS and HA content had an effect on the compression properties and porosity of the scaffolds. Increasing HA content weakened the strength from 31.34 MPa (W3H3) to 26.83 MPa (W3H15) but increased the modulus and porosity from 598.09 MPa and 47.8% to 721.97 MPa and 63.3%, respectively. The larger porosity was able to provide more room for cell ingrowth. Meanwhile, a larger modulus meant a larger anti-deforming capacity. When WS content was increased to 8 wt% (W8H3), both the compression strength and modulus reached a climax of 35.72 MPa and 883.22 MPa, indicating enhanced loading and anti-deforming capacity. In general, the scaffolds possessed a mudulus between 500 and 900 MPa, which could meet the requirements of trabecular bone (10–2000 MPa)^46^. In literature^34^, PLA/15 wt%WS composite scaffold with 64.17% of porosity possessed only about 7.97 MPa of compressive yield strength and 30.7 MPa of modulus. These tensile values of PLA/15 wt%WS composite scaffold were much lower than those of W15H3. In other words, the addition of HA enhanced dramatically the anti-deforming capacity of PLA/WS composite scaffold. Therefore, PLA/WS/HA composite scaffold would be more suitable to be applied in TE.

**Table 4:** Compression properties of PLA/WS/HA scaffolds.

Sample	Compression (yield)		Porosity	
	Strength	Modulus		
W3H3	31.34 ± 0.16	598.09 ± 15.24	47.8 ± 1.2	
W3H8	32.15 ± 0.44	506.01 ± 3.52	52.1 ± 0.6	
W3H15	26.83 ± 0.08	721.97 ± 18.27	63.3 ± 0.4	
W8H3	35.72 ± 0.97	883.22 ± 12.32	55.7 ± 0.9	
W15H3	27.58 ± 1.08	723.80 ± 10.23	61.7 ± 0.5	

## Conclusion

In this work, poly(lactic acid)/walnut shell/hydroxyapatite (PLA/WS/HA) composite filaments were prepared and processed by using fused deposition (FDM) modeling technology. HA was treated with silane and resulted in the formation of Si–O–P groups, which was benefitial to enhance interfacial compatibility between HA and PLA. The evaluation of the thermal properties showed that incorporating either HA or WS improved the thermal stability of PLA. The onset and maximum degradation rate temperatures were improved from 299.4 °C and 339.2 °C to 322.5 °C and 361.96 °C, respectively. Mechanical tests showed that the tensile and compression strength of PLA were reduced to 34.24 MPa (W15H3) and 75.74 MPa (W3H15). The toughening mechanism analysis showed that the PLA matrix was toughened by fillers, resulting in a higher tensile elongation (W8H3, 6.76%). The water uptake results showed that all the moisture contents increased with the increase of HA or WS content, indicating an improved hydrophilia. The water soaking obviously dropped the tensile and compression strengths. Finally, scaffolds with porosities of 47–62%, interconnected pores with diameters of 1.2 mm and moduli of 500–900 MPa were manufactured by using FDM. These results indicated that the PLA/WS/HA composite filaments have the potential application prospects in structural components such as bone implants.

## Data Availability

All data generated or analyzed during this study are included in this article (and its supplementary information files).
